# Inhaled H_2_ or CO_2_ Do Not Augment the Neuroprotective Effect of Therapeutic Hypothermia in a Severe Neonatal Hypoxic-Ischemic Encephalopathy Piglet Model

**DOI:** 10.3390/ijms21186801

**Published:** 2020-09-16

**Authors:** Viktória Kovács, Gábor Remzső, Valéria Tóth-Szűki, Viktória Varga, János Németh, Ferenc Domoki

**Affiliations:** Department of Physiology, University of Szeged Faculty of Medicine, H-6720 Szeged, Hungary; remzso.gabor@med.u-szeged.hu (G.R.); toth-szuki.valeria@med.u-szeged.hu (V.T.-S.); varga.viktoria.eva.22@gmail.com (V.V.); acetilkolin07@hotmail.com (J.N.); domoki.ferenc@med.u-szeged.hu (F.D.)

**Keywords:** hypoxic-ischemic encephalopathy, perinatal asphyxia, therapeutic hypothermia, piglet model, hydrogen ventilation, brain-derived neurotrophic factor

## Abstract

Hypoxic-ischemic encephalopathy (HIE) is still a major cause of neonatal death and disability as therapeutic hypothermia (TH) alone cannot afford sufficient neuroprotection. The present study investigated whether ventilation with molecular hydrogen (2.1% H_2_) or graded restoration of normocapnia with CO_2_ for 4 h after asphyxia would augment the neuroprotective effect of TH in a subacute (48 h) HIE piglet model. Piglets were randomized to untreated naïve, control-normothermia, asphyxia-normothermia (20-min 4%O_2_–20%CO_2_ ventilation; T_core_ = 38.5 °C), asphyxia-hypothermia (A-HT, T_core_ = 33.5 °C, 2–36 h post-asphyxia), A-HT + H_2_, or A-HT + CO_2_ treatment groups. Asphyxia elicited severe hypoxia (pO_2_ = 19 ± 5 mmHg) and mixed acidosis (pH = 6.79 ± 0.10). HIE development was confirmed by altered cerebral electrical activity and neuropathology. TH was significantly neuroprotective in the caudate nucleus but demonstrated virtually no such effect in the hippocampus. The mRNA levels of apoptosis-inducing factor and caspase-3 showed a ~10-fold increase in the A-HT group compared to naïve animals in the hippocampus but not in the caudate nucleus coinciding with the region-specific neuroprotective effect of TH. H_2_ or CO_2_ did not augment TH-induced neuroprotection in any brain areas; rather, CO_2_ even abolished the neuroprotective effect of TH in the caudate nucleus. In conclusion, the present findings do not support the use of these medical gases to supplement TH in HIE management.

## 1. Introduction

Hypoxic-ischemic encephalopathy (HIE) is defined as acute/subacute brain injury elicited by asphyxia in the perinatal period [1,2]. The hallmarks of perinatal asphyxia include severe systemic hypoxia, hypercapnia, acidosis, and/or reduced cerebral blood flow. HIE is diagnosed based on the clinical presentation of encephalopathy and laboratory findings of recent asphyxia often showing multi-organ involvement in hypoxic-ischemic injury [3,4]. HIE is a major cause of neonatal death and long-term disability worldwide and the medical costs of HIE management can be expressed in billions of dollars in developed countries alone [5,6]. Therefore, neuroprotective therapies reducing brain injury and also preventing multi-organ failure are urgently needed, even though mild whole-body hypothermia treatment (therapeutic hypothermia; TH) has been successfully implemented into the clinical practice and was shown to improve long-term outcome measures [1]. However, TH alone is clearly insufficient for the successful treatment of all HIE patients; on average, seven infants affected with severe HIE must be treated with TH to reduce by one the number of deaths or major disabilities [7]. Therefore, further preclinical research is warranted to identify additional treatment options that can complement the neuroprotection afforded by TH. The term newborn pig provides a suitable large-animal experimental model for translational HIE research as its gyrencephalic brain structure, brain developmental stage at birth, cerebral metabolic rates of glucose and oxygen are all comparable to term human babies [8,9,10]. Notably, TH was also shown to be effective in a piglet HIE model, lending support to the model’s translational value [11,12]. Among the many putative neuroprotective therapies, our research group focuses on inhaled neuroprotective gases as they offer a simple, straightforward administration route that is feasible even under clinical conditions, as acute management of severely asphyxiated babies often must include intratracheal intubation and mechanical ventilation. In the present study, we assessed two previously reported neuroprotective gases: H_2_ and CO_2_.

Ohsawa et al. reported in their seminal paper that inhaled H_2_ was neuroprotective in an adult rat stroke model [13], and it was also found to be effective in a rat HIE model as well [14]. Our research group was the first to show H_2_-induced neuro-vascular protection in a piglet acute HIE model [15], and then we characterized the beneficial effects of H_2_. We showed preserved neuro-vascular reactivity [16], reduced neuronal injury in most brain regions studied, improved recovery of EEG [17], reduced oxidative deoxyribonucleic acid (DNA) damage, limited microglial activation, and abolishment of asphyxia-induced increases in neuronal cyclooxygenase-2 (COX-2) immunopositive neurons [18]. However, an additive neuroprotective effect of H_2_ and TH has not yet been demonstrated. Indeed, a recent study investigated this in a piglet model [19]; however, there were no differences between the TH and the TH + H_2_ treated animals in the neurological/neuropathological scores. A potential cause for this finding was that all animals undergoing TH or TH + H_2_ fully recovered by the end of the observation period, excluding the possibility to detect such differences in treatment efficacy [19]. 

Hypercapnia is a cardinal feature of asphyxia; however, postasphyxial CO_2_ levels also play an important role in determining HIE outcome: hypocapnia was found to be an independent risk factor for severe neurodevelopmental disability and death [20]. Furthermore, inhalation of CO_2_ immediately after experimental asphyxia, leading to a so-called graded restoration of normocapnia, was shown to exert beneficial effects like reduction of seizure burden [21] and preservation of cerebral oxygenation [22].

Brain-derived neurotrophic factor (BDNF) is a member of the neurotrophic factor family that is highly expressed in the developing brain and plays an important role in regulating neural proliferation, differentiation, and survival [23,24]. Upregulation of BDNF has been implied to exert a neuroprotective effect in the hippocampus of newborn piglets following hypoxic stress [25]. Furthermore, BDNF upregulation was shown to be involved in TH-induced neuroprotection following cardiac arrest in the adult rat brain [26], and it was also observed in newborn piglets exposed to TH after hypoxic/ischemic stress [27]. BDNF has anti-apoptotic effects by blocking both caspase-independent and dependent pathways acting on caspase-3 and apoptosis-inducing factor (AIF), respectively. Although these genes were assessed in hypoxic or hypoxic-ischemic piglet models [25,27], the effect of asphyxia on the gene expression patterns has not yet been explored.

Therefore, the major objective of the present study was to assess whether there was an additive neuroprotective effect of H_2_ or CO_2_ when combined with TH compared to TH alone. To enhance the translational value of our study, we modified our previously published piglet HIE model [17] by eliciting asphyxia with lower FiO_2_ (4% instead of 6% O_2_) to represent more severe HIE patients that would especially benefit from combined therapies and provided the current state-of-the-art HIE management including TH. We also wished to start exploring the molecular mechanisms of neuronal injury at the gene expression level; therefore, we studied expression changes in BDNF, caspase-3, and AIF. We demonstrate that H_2_ or CO_2_ could not enhance the neuroprotective effect of TH in any brain areas assessed with either neuropathology or electrophysiology. We also made novel observations on how TH modifies gene expression after asphyxia in our model that may contribute to the neuroprotection. 

## 2. Results

**Figure 1:**
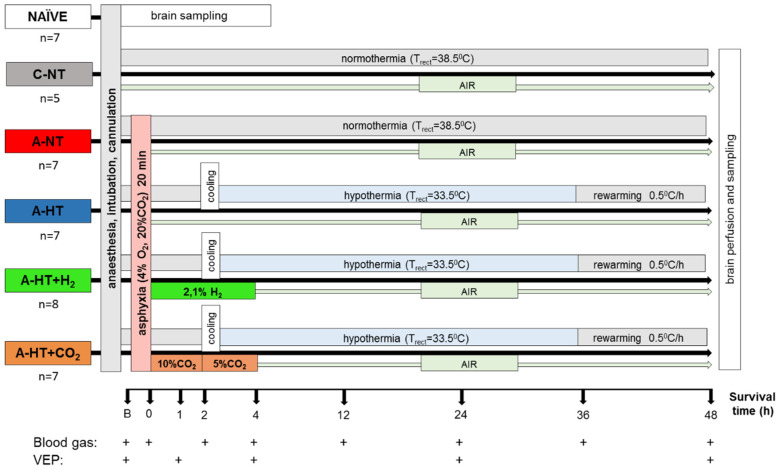
Overview of the experimental protocol. Untreated animals served as naïve controls for neuropathology and gene expression studies; piglets in the control-normothermia group (C-NT) were anesthetized, ventilated, and monitored but not subjected to asphyxia. Animals of the asphyxia-normothermia group (A-NT) were exposed to 20 min asphyxia induced by ventilation with 4% O_2_–20% CO_2_ gas mixture. Animals in the asphyxia-hypothermia group (A-HT) were cooled down to 33.5 °C, starting 2 h after reventilation, and were gradually rewarmed from 36 h to normothermia by 0.5 °C/h in 10 h. H_2_-treatment was initiated at the onset of reventilation after asphyxia (21%O_2_–2.1% H_2_, 4 h) and was combined with hypothermia (A-HT + H_2_ group). In a similar fashion, CO_2_-treatment was initiated at the onset of reventilation after asphyxia and was combined with hypothermia (A-HT + CO_2_ group). In order to achieve graded restoration of normocapnia, the animals were ventilated first with 21% O_2_–10% CO_2_ for 2 h and then with 21% O_2_–5% CO_2_ for 2 h before switching back to air. Arterial blood samples were collected and visual evoked potential (VEP) measurements were performed at the marked time points.

To address the major study objectives, five groups of anesthetized, instrumented animals were used, and control brain samples were obtained from naïve (untreated control) animals as well (Figure 1). Mortality of the applied asphyxia/HIE protocol was 12% (4/33): three piglets expired during asphyxia before they could have been assigned to any treatment group, and only one piglet in the asphyxia-hypothermia group was lost at 32 h of survival. Data from these animals were not included in the study.

### 2.1. Physiological Parameters during Asphyxia and HIE Development

**Figure 2:**
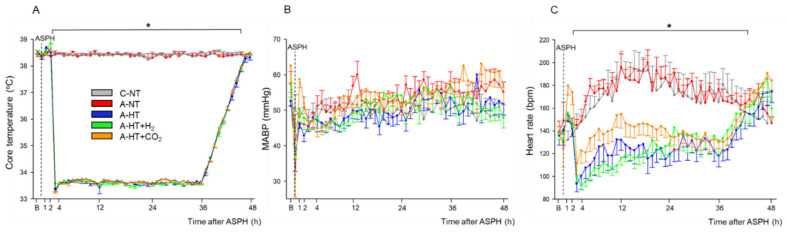
Core temperature (**A**), mean arterial blood pressure (MABP) (**B**), and heart rate (**C**) data in the time controls and in the asphyxia groups during baseline (B), in the last minute of 20 min asphyxia (ASPH), and the 48 h survival period. The core temperature and the heart rate values were significantly higher in the normothermic groups (control-normothermia C-NT and asphyxia-normothermia A-NT) compared to the hypothermic ones (asphyxia-hypothermia A-HT, A-HT + H_2_, and A-HT + CO_2_); however, there were no significant differences in MABP. Data are shown as mean ± SEM. *p* < 0.05 * normothermia vs. hypothermia groups.

At the beginning of the experiments, piglets in all groups had similar physiological values [28] of core temperature (38.5 ± 0.2 °C), mean arterial blood pressure (MABP; 57 ± 10 mmHg), and heart rate (HR; 140 ± 20 bpm), respectively (Figure 2A–C). Immediately after the onset of asphyxia, MABP and HR were first markedly raised (at peak, they were 73 ± 12 mmHg and 206 ± 22 bpm, respectively) but then continuously decreased by the end of the asphyxia (at the nadir, they were 38 ± 17 mmHg and 138 ± 33 bpm, respectively). Reventilation resulted in quick restoration of MABP and HR; then, MABP values were kept in the physiological range and the values did not differ significantly among the experimental groups throughout the 48 h observation period (Figure 2B). After the onset of the TH treatment, core temperatures differed between the normothermic and hypothermic animals (Figure 2A); also, a clear reduction in HR developed in the hypothermic groups of animals compared to the normothermic ones (Figure 2C).

**Figure 3:**
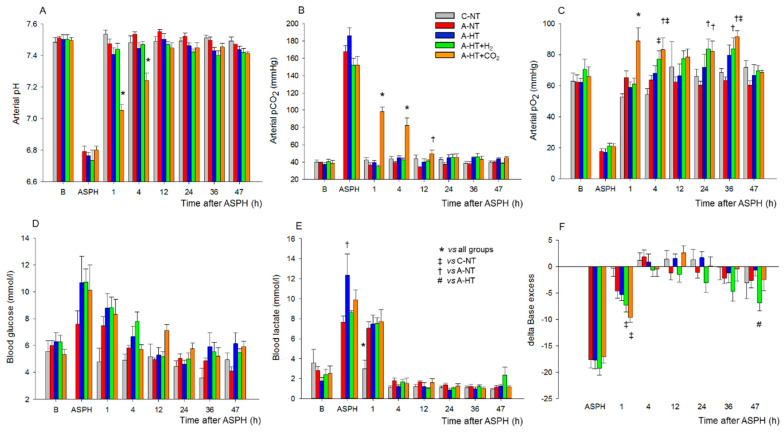
Blood chemistry data at baseline (**B**), in the last minute of 20 min asphyxia (ASPH), and the subsequent observation period. Arterial blood gas analysis revealed that asphyxia resulted in severe acidosis (**A**), hypercapnia (**B**), hypoxemia (**C**). Plasma glucose (**D**) and lactate levels (**E**) were markedly elevated along with large drops in base excess (**F**), indicating the metabolic response to asphyxia. After asphyxia, arterial pH and pCO_2_ values reflected the effect of 10–5% CO_2_ ventilation in the graded reduction of normocapnia group (A-HT + CO_2_); otherwise, reventilation restored most of the parameters by 4 h, and they were not significantly different from baseline levels afterwards. Experimental groups: control-normothermia (C-NT), asphyxia-normothermia (A-NT), asphyxia-hypothermia (A-HT), A-HT supplemented with H_2_ (A-HT + H_2_) or CO_2_ (A-HT + CO_2_), respectively. Bars and whiskers represent mean ± SEM, *p* < 0.05 * vs. all groups; ^‡^ vs. C-NT; ^†^ vs. A-NT; ^#^ vs. A-HT.

The monitored blood chemistry parameters such as arterial blood pH, blood gases, glucose, and lactate levels were similar and within the normal range in all groups at baseline (Figure 3A–F). However, blood analysis at the end of asphyxia revealed severe combined metabolic and respiratory acidosis with corresponding hypercapnia and lactacidemia (Figure 3A,B,E), as well as hypoxemia (Figure 3C) and negative base excess (Figure 3F), that were similar in all groups exposed to asphyxia. After asphyxia, blood gases quickly returned to baseline levels and then they were not statistically different from the corresponding values of the control-normothermia group, with the notable exception of the asphyxia-hypothermia+CO_2_ group. In this group, the continued ventilation with 10% or 5% CO_2_ was reflected in the gradual restoration of pH and pCO_2_ at 1 and 4 h, respectively (Figure 3A,B). The elevated blood lactate levels also gradually returned to baseline levels; they were still significantly elevated at 1 h after asphyxia in all groups compared to the control-normothermia group, but there was no difference among the groups at any time point afterwards (Figure 3E). The blood glucose levels remained slightly elevated at 1 h of reventilation but then returned to baseline values by 4 h of reventilation (Figure 3D).

#### Brain Interstitial H_2_ Concentration Measurements

**Figure 4:**
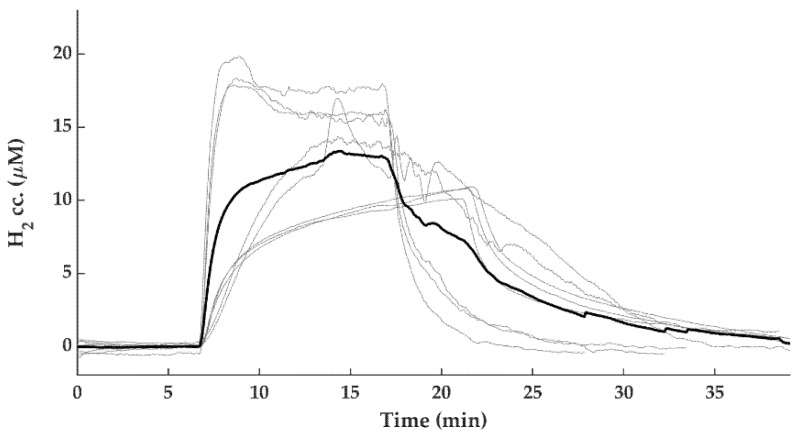
Cerebrocortical H_2_ concentration changes during 2.1% H_2_ ventilation. H_2_ gas was ventilated to the animals (*n* = 8 trials from *n* = 3 piglets) until steady state H_2_ levels were reached at 10–15 min after initiation of H_2_ ventilation (grey lines—individual traces, bold black line—mean).

To show the efficacy and the dynamics of the applied H_2_-inhalation protocol to increase brain H_2_ levels, cerebrocortical H_2_ levels were determined in three additional normothermic, normoxic control animals, and they were found to increase quickly and reach steady-state levels in 10–15 min; the average H_2_ concentration was 13.1 ± 3.4 µM (Figure 4).

### 2.2. Electroencephalography (EEG) Analysis

**Figure 5:**
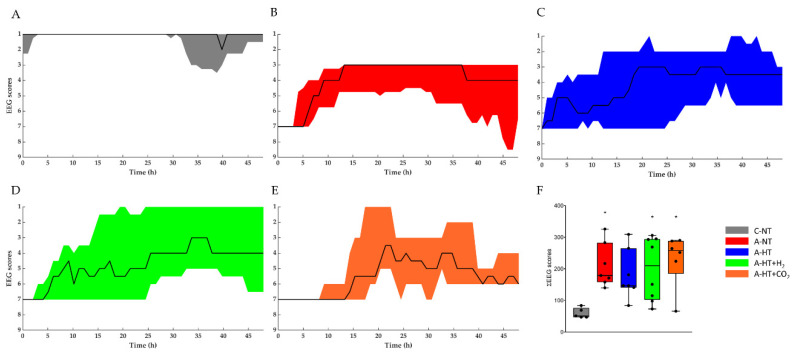
The EEG recordings were scored using a previously published [14] amplitude-based scoring system (Table S1), where higher scores represent progressive deterioration of electrical activity. Experimental groups: control-normothermia (C-NT), asphyxia-normothermia (A-NT), asphyxia-hypothermia (A-HT), A-HT supplemented with H_2_ (A-HT + H_2_) or CO_2_ (A-HT + CO_2_), respectively. (**A**–**E**): The black line shows the median values whereas the shaded area represents the 25th-75th interquartile range. C-NT animals were characterized by continuous EEG activity and low EEG scores during the entire experiment, whereas asphyxia induced isoelectric EEG that was followed by slow regeneration of electrical activity in all experimental groups in the observation period. There were no significant differences among the treatment groups at any time points; however, the large intra-individual variability can be appreciated. (**F**): The sum of EEG scores during the observation period in the experimental groups. The black line represents the median, the box the interquartile range, the whiskers the 10th-90th percentiles, the bullets the raw values. The summated EEG scores were significantly higher in the A-NT, A-HT + H_2_, and A-HT + CO_2_ asphyxia groups compared to the C-NT (* *p* < 0.05) group, but this was not the cause in the A-HT group, suggesting that the best outcome was found in these animals.

At baseline conditions before asphyxia, similar continuous EEG activity (>25 μV amplitude) could be observed in all groups. The control-normothermia group maintained continuous, high-amplitude EEG activity throughout the observation period (Figure 5A). The EEG became isoelectric within 1–2 min after the onset of asphyxia; then, the EEG activity regenerated progressively but showed large inter-individual variability (Figure 5B,E). The total scores showed well the effect of asphyxia, but there were no significant differences among the treatment groups (Figure 5F). 

**Figure 6:**
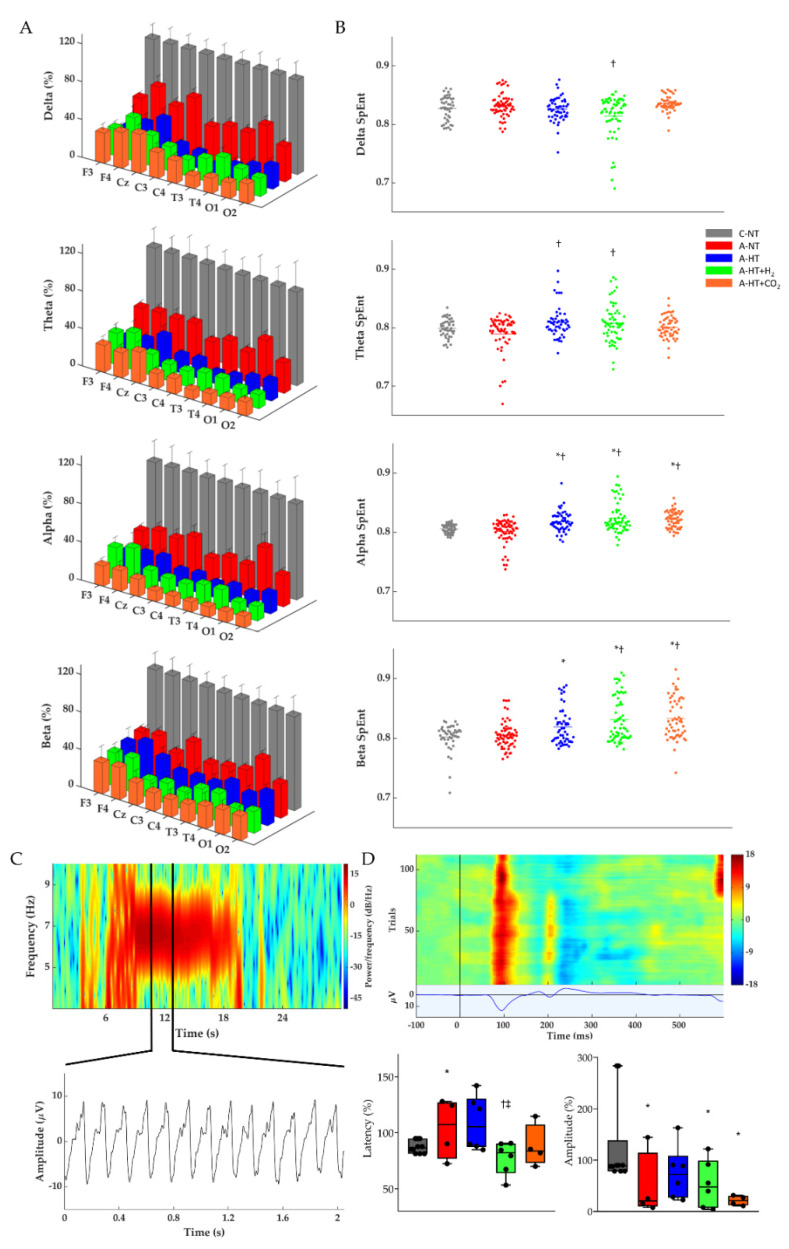
(**A**) Average power spectral density (PSD) changes in each experimental group for the four main frequency bands at the end of the 48 h observation period. All PSDs were normalized to their respective control values for each lead. Delta, theta, alpha, and beta PSDs across the different leads show significant differences between each group. (**B**) Corresponding individual spectral entropy (SpEnt) values in each group. (**C**) Representative spectrogram of a generalized seizure in a piglet from the asphyxia-normothermia group (~7 Hz) which could be detected by all 9 leads. Below the spectrogram, we highlighted a 2 s-long spike-and-wave seizure waveform. (**D**) Representative heat map of the recorded VEP trials with their average VEP waveform below. The two box plots show the latency and peak amplitudes of the P100 components at the 48 h time point in each group, normalized to the respective pre-asphyxia baseline values. The black line represents the median, the box the interquartile range, the whiskers the 10th–90th percentiles, the bullets the raw values. Experimental groups: control-normothermia (C-NT), asphyxia-normothermia (A-NT), asphyxia-hypothermia (A-HT), A-HT supplemented with H_2_ (A-HT + H_2_) or CO_2_ (A-HT + CO_2_), respectively. Data are mean ± SD; * vs. C-NT, ^†^ vs. A-NT, ^‡^ vs. A-HT, *p* < 0.05.

The restoration of EEG activity in the different groups was also quantitatively analyzed after rewarming toward the end of the 48 h observation period by determining power spectral density (PSD; Figure 6A, Table S2), spectral entropy (SpEnt; Figure 6B), and visual evoked potentials (VEP; Figure 6D). The effect of asphyxia on PSD in all frequency ranges is conspicuous (Figure 6A). Although the asphyxia-normothermia group shows higher PSDs, especially at lower (δ-θ) frequencies, than the hypothermia-treated groups, this was found to be due to the higher incidence of generalized seizures, abnormal waveforms, and spikes; for example, see Figure 6C—these seizures were virtually absent in the hypothermia-treated groups. SpEnt revealed high temporal complexity in all frequency bands that were due to the anesthesia alone in the control-normothermia group, while they were affected by asphyxia-induced changes in the other four groups. Comparing these, SpEnt values were increased in the hypothermia-treated groups compared to the asphyxia-normothermia group, especially at the higher frequency (α-β) ranges (Figure 6B). Furthermore, the lower temporal complexity shown by SpEnt in the asphyxia-normothermia group is also confirmed by the calculated lowest instantaneous spectral entropy (InstSpEnt) representing the whole EEG signal determined for this group. InstSpEnt values were 0.684 ± 0.054, 0.734 ± 0.073 *, 0.699 ± 0.69, and 0.723 ± 0.075 * for asphyxia-normothermia, asphyxia-hypothermia, asphyxia-hypothermia+H_2_, and asphyxia-hypothermia+CO_2_ groups, respectively (* *p* < 0.05, significantly different from the asphyxia-normothermia group). VEP waveforms were severely affected by asphyxia; the changes were dominated by attenuation of the P100 component amplitude. Compared to the respective baseline (pre-asphyxia) values, attenuation of the P100 component was significant in all groups exposed to asphyxia except for the asphyxia-hypothermia group (Figure 6D).

### 2.3. Neuropathology

**Figure 7:**
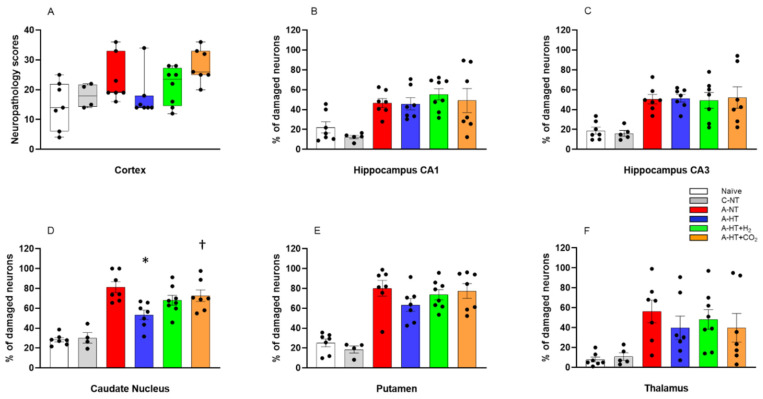
Neuronal injury evaluated at 48 h after asphyxia in the different cortical and subcortical regions. (**A**): In the neocortex, neuropathology scores suggested that asphyxia-induced neuronal injury was mitigated by hypothermia; however, there were no statistically significant differences among the groups (lines, boxes, whiskers, and bullets represent the median, the interquartile range, the 10th-90th percentiles, and raw data, respectively). (**B**–**F**): In the other assessed regions, cell counting revealed no significant differences in the low percentage of damaged neurons between the naïve and control-normothermia (C-NT) groups. Compared to the C-NT group, severe neuronal damage was detected in virtually all areas in the groups that were subjected to asphyxia. Neuronal damage was similar in both the CA1 and the CA3 hippocampal subfields in all groups exposed to asphyxia, despite hypothermia treatment. However, in the asphyxia-hypothermia (A-HT) group, a significant decrease in percentage of damaged neurons in the caudate nucleus was observed compared to the asphyxia-normothermia (A-NT) group, and there was a similar tendency in the putamen and the thalamus. Importantly, co-treatment with H_2_ or CO_2_ to achieve graded restoration of normocapnia did not augment the hypothermia-induced neuroprotection (A-HT + H_2_ and A-HT + CO_2_ groups, respectively). Instead, a significant increase in neuronal damage was observed in the caudate nucleus in the A-HT + CO_2_-treated group compared to the A-HT group. Data are mean ± SEM; bullets represent raw data, * vs. A-NT; † vs. A-HT, *p* < 0.05; statistical significance from naïve or C-NT groups is not shown. Representative photomicrographs are shown in Supplementary Figures S1–S5.

Neuropathology assessment confirmed severe HIE development induced by asphyxia, shown by increased neuronal damage compared to naïve or control-normothermia animals, in virtually all studied brain regions. TH appeared to mitigate the neuronal damage in the neocortex, the caudate nucleus, the putamen, and the thalamus; however, statistically significant neuroprotection was detected only in the caudate nucleus. In contrast, TH could not affect the severe neuronal damage in the hippocampus. Most importantly, the additional treatments with H_2_ and CO_2_ did not provide any additional beneficial effects; in contrast, in several regions, such as the neocortex or the basal ganglia, they appeared to abolish the neuroprotective effect of TH. In fact, neuronal damage in the caudate nucleus was significantly higher in the asphyxia-hypothermia+CO_2_ group compared to the asphyxia-hypothermia group (Figure 7).

### 2.4. Gene Expression Studies

**Figure 8:**
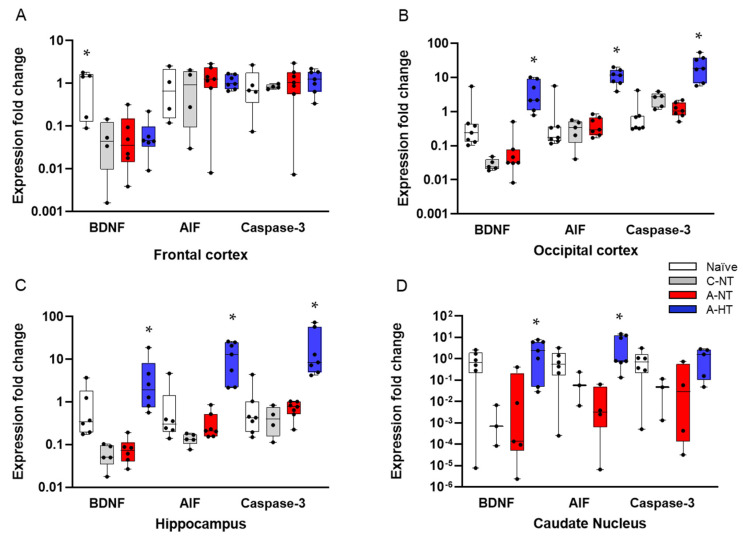
Gene expression data for BDNF, AIF, and caspase-3 genes in four different brain regions. (**A**): In the frontal cortex, BDNF mRNA levels were significantly higher in the naïve (untreated) group, but they were very similar in the control-normothermia (C-NT), asphyxia-normothermia (A-NT), and asphyxia-hypothermia (A-HT) groups. AIF and caspase-3 levels were also very similar in all four groups. (**B**): In the occipital cortex, the mRNA levels of all three genes were significantly elevated in the A-HT group compared to the A-NT group. (**C**): In the hippocampus, an identical pattern of gene expression was observed. (**D**): In the caudate nucleus, BDNF and AIF but not caspase-3 mRNA levels were significantly increased in the A-HT group compared to the A-NT group. Lines, boxes, whiskers, and bullets represent the median, the interquartile range, the 10th–90th percentiles, and raw data, respectively. * vs. A-NT *p* < 0.05.

Based on the neuropathology data, we selected four brain regions—the frontal cortex, the occipital cortex, the hippocampus, and the caudate nucleus—that differed from each other both in the degree of neuronal injury after asphyxia and the efficacy of TH to exert neuroprotection. We determined changes in BDNF, AIF, and caspase-3 expression to assess the impact of asphyxia and TH (Figure 8). There were no significant differences in the mRNA levels of any genes in any region between the control-normothermia and asphyxia-normothermia groups. Furthermore, as compared with the asphyxia-normothermia group, mRNA levels for all three genes remained unchanged in the frontal cortex in the asphyxia-hypothermia animals (Figure 8A). However, in the other three regions, BDNF and AIF mRNA levels were significantly increased in the asphyxia-hypothermia group compared to the asphyxia-normothermia animals (Figure 8B–D). Caspase-3 mRNA levels were also induced by hypothermia after asphyxia in the occipital cortex and the hippocampus (Figure 8B,C) but not in the caudate nucleus (Figure 8D). 

## 3. Discussion

The major findings of the present study are the following: (1) we employed a severe HIE model in which TH alone was unable to prevent severe neuronal injury; (2) we determined InstSpEnt and SpEnt in addition to PSD and VEP to characterize electrophysiological changes during HIE-development in our piglet model; (3) we made novel observations on region-specific gene expression changes induced by asphyxia or TH, starting to decipher the underlying mechanisms of the region-dependent neuroprotective effect of TH, and (4) we found that neither molecular H_2_ nor graded restoration of normocapnia with CO_2_ could provide an additive neuroprotective effect to TH in this piglet HIE model.

In the present study, we employed a severe HIE piglet model to represent the group of HIE patients in which TH alone is unlikely to prevent the development of adverse neurological consequences. These affected infants would benefit the most from adjunct neuroprotective therapies complementing the neuroprotective effect of TH. Two such promising adjunct medical gas therapies, namely H_2_ ventilation and graded restoration of normocapnia achieved with CO_2_ ventilation, have been tested in the present study. Hypothermia was initiated 2 h after asphyxia, in agreement with previous studies using hypothermia-induced neuroprotection in different HIE piglet models [29,30]. The 48 h observation period allowed most of the asphyxia-induced neuronal damage to develop [30]; it also enabled us to perform the recommended slow rewarming protocol (ΔT/t = 0.5 °C/h, 10 h) to prevent adverse systemic effects of cooling. However, neuronal damage may continue beyond the observed period, and the 34 h duration of therapeutic level TH employed in the present study may have been still suboptimal to exert the maximal neuroprotective effect of TH. This is a clear limitation of our study; indeed, neuroprotection afforded by 72 h local brain cooling was clearly superior to 48 h cooling in prenatal sheep [31]. Furthermore, the 2 h delay in hypothermia treatment initiation used in the present study may have also limited the neuroprotective effect of TH, although this would have likely facilitated the detection of potential additive effects as these were initiated immediately after asphyxia in the present study. These limitations should be considered when designing future studies to assess new treatments combined with TH.

EEG recording was used to determine a host of parameters aimed to characterize the functional consequences of asphyxia and to assess the efficacy of neuroprotective treatments in the present study. PSDs are easy to compute and clearly reflect the current functional state of the cortex as well as showing altered neuronal activity including seizures [32]. Studying entropy is more complicated due to the chaoticity and non-linearity of the biological (e.g., EEG) signal. InstSpEnt and SpEnt are relatively simple parameters that yield information on the instantaneous state of the whole signal and the normal power distribution in the respective frequency domain, respectively. Determination of InstSpEnt and SpEnt are commonly used approaches in electrophysiology [33,34] but not employed frequently in neonatology. In our study, we could show that, in the asphyxia-normothermia group, the higher PSD values were accompanied by lower InstSpEnt and SpEnt values, showing that low-entropy abnormal rhythmic (seizure) activities contributed more to the EEG signal than in the asphyxia-hypothermia groups. The higher InstSpEnt and SpEnt values measuring EEG signal complexity found in the hypothermia-treated groups are in concert with the neuroprotective effect of TH, even though hypothermia in this study was unable to restore PSDs after asphyxia. These findings help to establish novel outcome measures for translational HIE research or even the development of an artificial intelligence based toolbox for neonatal EEG monitoring. VEPs are also good predictors of neurological functional alterations in newborns [35,36]. Asphyxia resulted in abnormal VEP waveforms altering both signal latency and amplitude in all groups, similar to observations in animal models [37] or human neonates [38]. We observed significant amplitude decreases in all experimental groups; the smallest reduction was observed in the asphyxia-hypothermia group. All electrophysiology parameters (PSDs, InstSpEnt, SpEnt, VEP) indicated that the combination of hypothermia with either molecular H_2_ or graded restoration of normocapnia was not superior to hypothermia alone.

Neuropathology essentially confirmed our electrophysiology findings in the present study: asphyxia induced pronounced neuronal injury, especially in the hippocampus, the caudate nucleus, and the putamen, while neuronal damage was more modest in the neocortex. TH significantly reduced injury only in the caudate nucleus, in which region neuronal injury was most pronounced. Thus, in theory, our HIE model was sufficient to show whether the applied adjunct neuroprotective therapies could in fact facilitate hypothermia-induced neuroprotection to reach statistical significance. However, neither molecular H_2_ nor graded restoration of normocapnia with CO_2_ was able to facilitate neuroprotection; instead, in the caudate nucleus, the combination of TH with graded restoration of normocapnia significantly abolished hypothermia-induced neuroprotection. Similar tendencies were present in virtually every assessed region for both treatments. Our study has a clear limitation which we must declare: the observed statistical power of the multiple comparisons (ranging between 0.11 and 0.84) were low in some regions to be sure to avoid type II errors. However, we believe that this limitation may have only prevented us from not showing the adverse interaction of the adjunct treatments with TH, but a marked additive neuroprotective effect of these was not overlooked in any regions.

Molecular H_2_ has become an intensively studied cytoprotective/neuroprotective medical gas in the last decade; for recent reviews of the widespread beneficial effects of H_2_, please refer to [39,40,41,42]. Concerning HIE, many rodent studies using the Rice–Vannucci model of hypoxic-ischemic stress established the neuroprotective effect of H_2_ [14,43,44,45,46]. Our research group was the first to show the beneficial effect of H_2_ in a piglet HIE model, as H_2_ was found to preserve neurovascular function both in the acute (at 1–2 h) and in the subacute (at 24 h) after asphyxia [15,16]. Furthermore, using more severe asphyxia that was very similar to the one employed in the present study, H_2_ was shown to significantly reduce neuronal injury, oxidative stress, and neuroinflammation assessed also at 24 h after asphyxia [17,18]. In the present study, even more severe stress was employed (i.e., ventilation with 4% instead of 6% O2 during asphyxia). This level of hypoxia during asphyxia was chosen as we found that, if using 6% O2, hypothermia alone was so neuroprotective, this would have likely prevented the assessment of combined hypothermia+H_2_ treatment (unpublished observations). This potential limitation appears to have manifested in a recent study, in which both the hypothermia and the hypothermia+H_2_ treated piglets after asphyxia made a very similar, essentially full recovery, excluding the possibility for statistically valid comparisons between the efficacy of the treatments [19]. In the present study, we used an identical H_2_ administration as in our previous studies; namely, H_2_ treatment was applied in the first four hours of reventilation/reoxygenation. We now also showed for the first time using H_2_-selective microelectrodes that, in this experimental model, the applied H_2_ administration protocol results quickly (in 10 min) in steady-state interstitial brain H_2_ levels of ~10–20 μM, and these values are in accordance with blood H_2_ levels found in rats in which H_2_-induced neuroprotection against ischemic stress was demonstrated [13]. As we did not test cerebral H_2_ levels after asphyxia or during TH, the changes in cerebral blood flow in either condition could affect the kinetics of H_2_ level changes; this is also a clear limitation of the findings. However, the applied H_2_ administration route was shown to be appropriate for quick delivery of the putative neuroprotective gas in the immediate/early reoxygenation period after asphyxia. 

In contrast to H_2_, the neuroprotective actions of CO_2_ in HIE are less established. It is clear that, during hypoxic stress (8% O2 inhalation), low CO_2_ levels (hypocapnia) increase neuronal damage in the Rice–Vannucci rat model compared to mild hypercapnia, corresponding to 6–9% CO_2_ inhalation [47]. However, in a subsequent study, a detrimental effect of more severe hypercapnia (12–15% CO_2_ inhalation) has been demonstrated [48]. In our experiments, the concentration of inhaled CO_2_ was 20% to achieve pCO_2_ values corresponding with those recorded in “natural” birth asphyxia in piglets and humans [49,50,51]. This severe hypercapnia likely rather contributes to neuronal damage during asphyxia in our model at least in part through the severe cerebral acidosis that exceeds the systemic (blood) acidosis by more than 0.8 pH unit (6.79 vs. 5.94 for blood and brain extracellular pH), respectively [52]. Hypocapnia has been shown to be detrimental to the brain not only during hypoxic/ischemic stress but also in the resuscitation/reoxygenation period [20,53]. Hypocapnia may signal impaired restoration of cellular metabolism, predicting unfavorable outcome of resuscitation efforts [54]. Furthermore, hypocapnia can be a consequence of relative hyperventilation in response to metabolic acidosis, and prevention of hypocapnia by inhalation of 5%CO_2_ may enhance the efficacy of TH [55]. The question of whether increased CO_2_ levels (hypercapnia) maintained while establishing normoxia after asphyxia would convey neuroprotection has been proposed by a set of rat studies employing asphyxia very similar to the one employed in piglets in the present study [19,20,54]. In these studies, the graded restoration of normocapnia has been shown to enhance endogenous protecting mechanisms, preventing rapid restoration and overshoot of cerebral pH and improving oxygenation, importantly also counteracting seizures, suggesting a profound neuroprotective effect.

There is no clear explanation for the lack of efficacy of the combined treatment of either H_2_ or graded restoration of normocapnia with TH, as their precise mechanism of action is unknown. As neither treatment was tested under normothermic conditions in this study, it is possible that they could have exerted neutral or even detrimental effects in this experimental paradigm. Alternatively, hypothermia, H_2_ or graded restoration of normocapnia may target the activation of similar or even the same pro-survival mechanisms, and either one may activate these alone as efficiently as combined with each other. For instance, TH, CO_2_, and even H_2_ are known to reduce neuronal excitability and metabolism, providing means for the occlusion of protective effects [55,56,57,58].

Asphyxia-induced changes in gene expression are well-known to affect neuronal viability. However, data from large animal models are quite limited, and the results from these studies do not necessarily confirm findings established in rodents. For example, phosphorylation of extracellular signal-regulated kinases (ERK) and protein kinase B (Akt) were shown to be protective in the brains of neonatal rodents, suggesting the activation of anti-apoptotic signaling pathways [26,59,60]. In contrast, in our previous piglet study, we clearly demonstrated that baseline activation levels of ERK and Akt are very high even under normoxic conditions in the perinatal period in newborn pigs, and these phosphorylation levels—although they can be modified by selective inhibitors of ERK and Akt kinases—remain unaffected after asphyxia [61]. Therefore, it is of great importance to re-study these molecular pathways in large animal models, as these differences can explain the translation block that often appears with neuroprotective mechanisms operating in small but not large animal brains.

BDNF, AIF, and caspase-3 mRNA levels were assessed in this study as these genes have been previously shown to be involved in neuronal injury/survival associated with HIE and TH in piglet models [25,27,62,63]. Postnatal stimulation of BDNF and its downstream elements with dietary supplements has been shown to promote cognitive development in piglets [62]. Furthermore, BDNF expression was upregulated by nicotine in the early phase (4 h) of HIE development triggered with hypoxia in piglets, suggesting therapeutic potential for nicotine [25]. Olson et al. found that BDNF mRNA was upregulated by the applied hypoxic/ischemic insult itself, and the response was unaltered by the cooling temperatures (35, 33.5, or 30 °C) employed in the study [27]. However, we observed that, compared to control-normothermic animals, BDNF mRNA levels were essentially the same as in the asphyxia-normothermia group. However, we did observe a significant increase in BDNF mRNA levels in the asphyxia-hypothermia group in three regions: the occipital cortex, the hippocampus, and the caudate nucleus. These findings suggest that hypoxia/ischemia and asphyxia may have different effects on BDNF expression in the piglet brain, and BDNF overexpression may have a role in the mechanism of hypothermia-induced neuroprotection. AIF and caspase-3 activation were involved in neuronal apoptosis in the frontal cortex following global cerebral ischemia, and expression of these factors was reduced by TH in minipigs [63]. Wang et al. reported that rewarming from hypothermia after HI was associated with apoptosis in the motor and piriform cortex in a piglet model [64]. Rapidly rewarmed piglets (4 °C/h) had more caspase-3 cleavage in cerebral cortex than did piglets that remained hypothermic or piglets that were rewarmed slowly (0.5 °C/h). Using a caspase-3 inhibitor, apoptosis was suppressed with rewarming. These results suggest that rewarming from hypothermia after HI may promote apoptosis through pathways involving caspases [64]. However, rapid or slow rewarming may have an indirect effect on the brain; a recent elegant study of prenatal lambs demonstrated that slow or rapid rewarming has no effect on neuronal survival if the hypothermia treatment is restricted to the brain, and there is no systemic hypothermia [65]. We found that TH after asphyxia upregulated both AIF and caspase-3 mRNA levels in the hippocampus, but only AIF and not caspase-3 in the caudate nucleus, coinciding with the selective neuroprotective effect of TH in the caudate nucleus. Our results are preliminary as they are limited to mRNA levels; therefore, no speculation about protein levels let alone activity should be assumed based on these findings alone. 

## 4. Materials and Methods 

### 4.1. Animals

Ethical approval and permission to conduct the animal experiments were obtained in a three-step process. First, the project (title: Study of neuroprotective strategies in a hypoxic-ischemic encephalopathy newborn pig model) was first reviewed and approved by the Institutional Animal Care and Use Committee of the University of Szeged (MÁB, project id nr.: I.74–7/2015, approved on 26 February 2015). Second, the project was forwarded to, reviewed, and approved by the Hungarian National Scientific Ethical Committee on Animal Experimentation (ÁTET). Third, based on the approval and recommendation by the ÁTET, the permit to obtain the animals was issued by the government agency National Food Chain Safety and Animal Health Directorate of Csongrád County, Hungary (permit nr: XIV./1414/2015, approved on 29 April 2015). The procedures were performed according to the guidelines of the Scientific Committee of Animal Experimentation of the Hungarian Academy of Sciences (updated Law and Regulations on Animal Protection: 40/2013. (II. 14.) Gov. of Hungary), following the EU Directive 2010/63/EU on the protection of animals used for scientific purposes and reported in compliance with the ARRIVE guidelines.

Newborn (<24 h old) male Landrace piglets (*n* = 48, weighing between 1.5 and 2.5 kg) were obtained from a local company (Pigmark Ltd., Co., Szeged, Hungary) and delivered to the laboratory on the morning of the experiments. The animals were anesthetized with an intraperitoneal injection of sodium thiopental (45 mg/kg; Sandoz, Kundl, Austria). The animals were placed on a servo-controlled heating pad (Blanketrol III, Cincinnati SUB-zero, Cincinnati, OH, USA), keeping their core temperature in the physiological range (38.5 ± 0.5 °C). The skin was disinfected, and the animals were intubated through a tracheotomy and then mechanically ventilated with warmed, humidified medical air (21% O_2_, balance N_2_) at a frequency of 30–35 breaths/min, using a pressure controlled ventilator applying peak inspiratory pressure = 120–135 mmH_2_O. Aseptic technique was followed during all aspects of the animal surgery. With the exception of the untreated naïve animals, the right carotid artery and femoral vein were cannulated with catheters. To maintain anesthesia/analgesia, the animals were given first a bolus injection of morphine (100 μg/kg; Teva, Petach Tikva, Israel) and midazolam (250 μg/kg; Torrex Pharma, Vienna, Austria) and then given a continuous infusion of morphine (10 μg/kg/h), midazolam (250 μg/kg/h), and fluids (5% glucose, 0.45% NaCl 3–5 mL/kg/h) through the femoral vein catheter. The right carotid artery catheter was placed for continuous monitoring of MABP and HR throughout the whole experiment. The wounds were closed and covered with warm compress to minimize heat and fluid losses. The animals were placed into a neonatal incubator (SPC 78-1; Narco Air-Shields, Inc., Hatboro, PA, USA). Oxygen saturation, mean arterial blood pressure (MABP), heart rate (HR), and electrocardiogram (ECG) were continuously monitored using a Hewlett-Packard M1094 monitor (Palo Alto, CA, USA) and recorded online (MecifView, Arlington, TX, USA). Core temperature was maintained rigorously at 38.5 ± 0.5 °C with a servo-controlled heating pad. Prophylactic antibiotics were given intravenously (penicillin: 50 mg/kg/12 h, Teva, Petah Tikva, Israel and gentamicin: 2.5 mg/kg/12 h, Sanofi, Paris, France) and the urinary bladder was tapped by suprapubic puncture every 12 h. Arterial blood gases, along with blood sugar and lactate levels, were determined (~300 μL/sample; EPOC Blood Analysis, Epocal Inc., Ottawa, ON, Canada) at baseline, at the end of asphyxia, and then at selected intervals up to 47 h to keep blood gas values in the physiological range during the survival period.

### 4.2. Experimental Protocol

#### 4.2.1. Electroencephalography (EEG)

**Figure 9:**
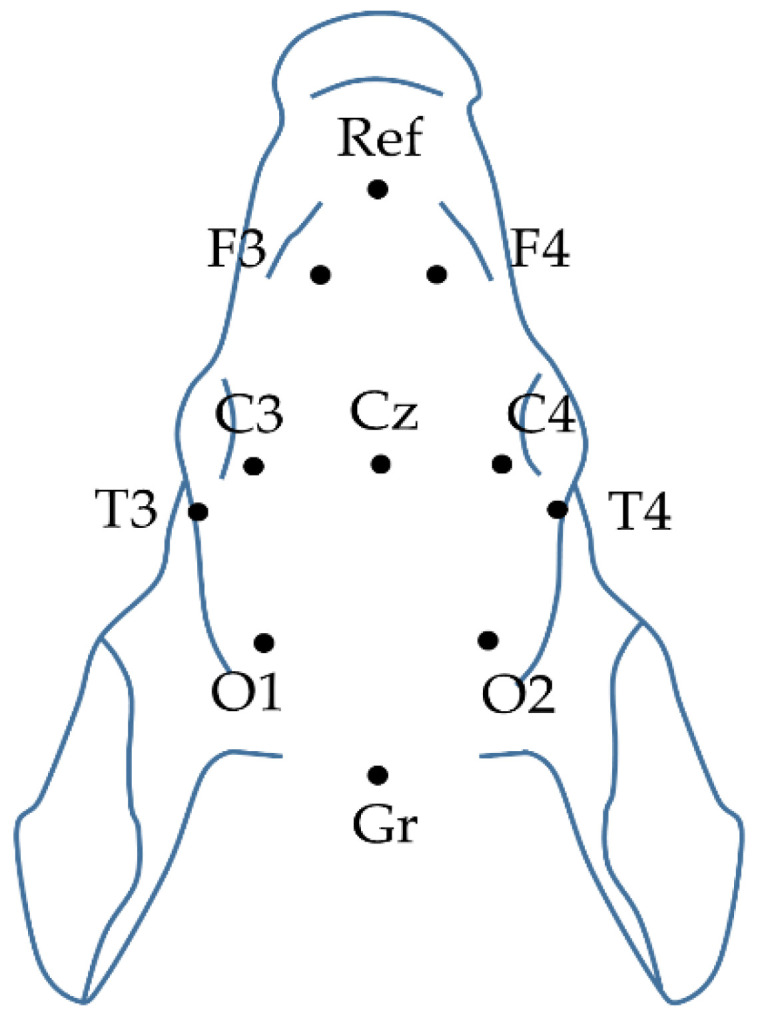
Plan view of the piglet scalp with the modified neonatal EEG montage according to the international 10–20 system.

EEG activity was recorded via subcutaneously inserted silver electroencephalograph (Natus Neurology, Middleton, WI, USA) electrodes above the fronto-parietal and occipital cortex with 256 Hz sampling rate (Figure 9). The impedance of all electrodes was checked to be below 5 kΩ. EEG signal was amplified (Nicolet EEG v32, Natus Medical Inc, San Carlos, CA, USA), recorded, and visualized online during the entire experiment with the manufacturer’s software (Nicolet One). Data were stored on a hard disc and were analyzed offline. Then, 10 min EEG intervals recorded at the beginning of each hour after asphyxia were scored using an amplitude-based scoring system (Table S1) [15,66]. Briefly, continuous and high-amplitude background activities (>10 μV) were given lower scores while severely depressed and isoelectric activities (<10 μV) received higher ones. In addition, if seizures appeared in the evaluated hour, 2 extra points were added. EEG monitoring was followed by the American Clinical Neurophysiology Society’s guidelines on continuous EEG monitoring in neonates [67].

Visual evoked potentials (VEP) were evoked with 1 Hz flash using a stroboscope. At least 100 trials were averaged in each animal for determining the stable average VEP waveform. Grand mean averages were calculated for the P100 peak’s (O_1_ and O_2_ electrodes) amplitude and latency changes. The process of evoking VEPs followed the American Clinical Neurophysiology Society’s guidelines on visual evoked potentials [68].

#### 4.2.2. EEG Spectral Analysis

The broad band signals were filtered, applying a bandpass filter (1–30 Hz). After signal decomposition (delta (1–4 Hz), theta (4–8 Hz), alpha (8–13 Hz), beta (13–30 Hz)), we calculated the PSDs. We used the fast Fourier transform (FFT) of the signal by applying a 30 s moving window with 1 s steps, using a Gaussian window. All PSD values were estimated by using the Welch’s method. The determined PSDs were summed, averaged, and normalized to the baseline level for each frequency band [32].

InstSpEnt and SpEnt calculations were based on Shannon entropy or information entropy [69]. The calculated Shannon entropy reflects the signal’s normal power distribution in the frequency domain. It is also commonly used to determine the depth of the anesthesia [70]. The SpEnt values were thresholded, setting the level to the standard deviation of the SpEnt (threshold = mean − SD). Thresholding was necessary due to the length of the signal (we did not use segmentation), averaging, and the quite narrow window sizes for each frequency band. The thresholded values were compared with the InstSpEnt. The SpEnt were presented as individual values for each group and channel. We also used Shannon entropy for seizure detection.

All data analysis was performed in the MATLAB (Mathworks Inc., Natick, MA, USA) environment with appropriate toolboxes [71], built-in functions, and custom written scripts. Plotting the SpEnt, we used a function written by Rob Campbell (2020), notBoxPlot (https://github.com/raacampbell/notBoxPlot), GitHub.

#### 4.2.3. Cortical H_2_ Concentration Measurements

In additional animals (*n* = 3), we performed craniotomy while the animal’s head was fixed in a stereotaxic frame. H_2_ microsensors (external tip diameter: 50 µm) were obtained from Unisense (Aarhus, Denmark). We mounted the electrode to a stereotaxic manipulator for the 2-point calibration. Before the calibration and the measurements, the electrode was polarized at +1000 mV for 1 h. The zero H_2_ reading was done in water phase, vigorously bubbled with N_2_ gas for at least 5 min in the CAL300 calibration chamber (Unisense, Aarhus, Denmark). Maximal H_2_ reading was done in water phase and calibration chamber as well, bubbled vigorously with a gas mixture containing 95% N_2_ and 5% H_2_. In this case, the H_2_ partial pressure was 0.05 ATM and the solubility was 794.64 µmol/L/atm at 22 °C. To calculate the maximal concentration, we multiplied the partial pressure with the solubility, which was 39.732 µM [72]. Data were recorded with 1 Hz sampling rate in SensorTrace Logger software (Unisense, Aarhus, Denmark). Calibrations were performed at room temperature; thus, during data analysis, we applied temperature correction.

### 4.3. Experimental Groups

Experimental protocol is outlined in Figure 1. Animals participating in the experiment were divided into 6 groups as follows. (1) Untreated animals served as naïve controls for neuropathology and gene expression studies (naïve, *n* = 7). After the surgical procedure, one hour of recovery period allowed stabilization of monitored physiological parameters prior obtaining baseline values. After recording the baseline physiologic parameters, animals were divided into 5 additional groups: (2) normoxic time controls (control-normothermia; *n* = 5) with 48 h of survival; (3) animals undergoing 20 min asphyxia (asphyxia-normothermia; *n* = 7); (4) asphyxia combined with mild whole body hypothermia (asphyxia-hypothermia; *n* = 7); (5) asphyxia combined with 4 h of hydrogen ventilation combined with mild whole body hypothermia (asphyxia-hypothermia+H_2_; *n* = 8); (6) asphyxia combined with 4 h of CO_2_ ventilation combined with mild whole body hypothermia (asphyxia-hypothermia+CO_2_; *n* = 7).

The piglets among the four asphyxia-exposed groups were randomized with two consecutive coin flips. Asphyxia in groups asphyxia-normothermia, asphyxia-hypothermia, asphyxia-hypothermia+H_2_, and asphyxia-hypothermia+CO_2_ was induced by ventilation with a hypoxic/hypercapnic gas mixture containing 4% O_2_ and 20% CO_2_ for 20 min while respiratory rate was reduced from 30 to 15 breaths/min, and intravenous glucose administration was suspended. Arterial blood gas was determined to check the severity of perinatal asphyxia at the 19th min of asphyxia. Piglets were reventilated (respiration rate; RR: 30 1/min) in asphyxia-normothermia and asphyxia-hypothermia groups with room air, whereas in the asphyxia-hypothermia+H_2_ group, this was achieved with a gas mixture containing hydrogen gas (2.1% H_2_, 21% O_2_, balance N_2_). After 4 h, hydrogen treatment was stopped and ventilation with room air was restored. In the asphyxia-hypothermia+CO_2_ group, piglets were reventilated with a normoxic hypercapnic gas mixture containing 10% CO_2_ for 2 h and thereafter 5% CO_2_ also for 2 h. Ventilation was continued with room air throughout the survival period.

In groups designated to receive hypothermia, we delayed the induction of hypothermia by 2 h to mimic clinical delays in inducing hypothermia. At 2 h after asphyxia, we induced whole-body hypothermia to a goal rectal temperature of 33.5 °C by using servo-controlled heating pad. Rectal temperature decreased to 33.5 °C in ~40 min, and rectal temperature has been found to track brain temperature within 0.2 °C in this model [66]. Piglets remained hypothermic for 34 h. At 36 h after asphyxia, we initiated whole-body rewarming at 0.5 °C/h by increasing the water temperature circulating through the warming blanket until normothermia (38.5 °C) was reached. All piglets were euthanized at 48 h after asphyxia. Both carotid arteries were catheterized in the distal direction and the brains were perfused through them with cold (4 °C) physiological saline. The brains were gently removed from the skull, and tissue samples (~100 mg) taken from the left hemisphere (frontal-, parietal-, temporal-, occipital cortex; hippocampus, caudate nucleus, putamen, thalamus) were snap frozen in liquid N_2_ and stored at −80 °C before total ribonucleic acid (RNA) isolation. The intact right hemispheres were immersion-fixed in 4 °C, 4% paraformaldehyde solution and further processed for histology after two weeks.

### 4.4. Histology

Paraffin-embedded, 4-μm sections were produced from the frontal, temporal, parietal, occipital lobes as well as the hippocampus CA1/CA3, thalamus, putamen, and nucleus caudatus areas using a microtome (Leica Microsystems, Wetzlar, Germany) and mounted on sylanized slides. Hematoxylin-eosin staining was performed to evaluate the extent of neuronal damage in the thalamus, putamen, nucleus caudatus, and the hippocampal CA1 and CA3 regions, which was assessed with manual cell counting by two independent observers in non-overlapping areas using ImageJ software (Wayne Rasband, NIH, Bethesda, MD, USA) (Figure 7B–F). Damaged neurons were identified using the major hallmarks of dark eosinophilic cytosol, as well as pyknotic or disrupted nuclei, by a researcher blinded to the experimental groups. The impact of asphyxia on subcortical brain regions was expressed as the percentage of damaged neurons.

In the cerebral cortex, however, neuropathology scores were determined (0–9) as described previously [15,16]. (Table 1). Briefly, the pattern of neuronal injury (none < scattered < grouped/laminar < panlaminar) was assessed in 40 non-overlapping fields of vision under 20× magnification with light microscopy (Leica Microsystems, Wetzlar, Germany) in each cortical region. Then, neuropathology scores (0–9) were given to each region based on the occurrence of the most severe pattern of injury observed. Thus, higher scores represented increasingly severe neuronal damage (Figure 7A).

### 4.5. Total RNA Extraction and cDNA Synthesis

Total RNA was extracted from homogenized brain tissues of the frontal and the occipital cortex, the hippocampus, and the caudate nucleus with Tri Reagent (Sigma, St. Louis, MA, USA) according to the manufacturer’s protocol. Briefly, 1 mL Tri Reagent was added to the homogenized samples. After 5 min incubation at room temperature to allow dissociation of nucleoprotein complexes, 0.2 mL of chloroform was added. The samples were mixed vigorously and then centrifuged at 12,000× *g* for 15 min at 4 °C. After centrifugation, the RNA was precipitated from the upper, colorless aqueous phase with 0.5 mL of isopropanol. The samples were then incubated at room temperature for 10 min and centrifuged at 12,000× *g* for 10 min at 4 °C. The supernatant was removed and the RNA pellet was washed once with 75% ethanol. The pellet was air dried and dissolved in diethyl pyrocarbonate (DEPC)-treated water. Total RNA quantity (OD260) and purity (OD260/280) were measured by a NanoDrop spectrophotometer (Thermo Scientific, Waltham, MA, USA)

### 4.6. Quantitative PCR Analyses

For quantitative PCR (qPCR), 1 μg of total RNA was reverse transcribed using the Maxima Reverse Transcriptase according to the manufacturer’s protocol with random hexamer priming (Thermo Fisher Scientific Inc. Waltham, MA, USA). qPCR was performed in a Bio-Rad CFX96 real-time system with the MaximaTM SYBR Green qPCR Master Mix (2X) (Thermo Fisher Scientific Inc. Waltham, MA, USA) and primers were designed specifically using the *Sus scrofa domestica* (white pig) Ensembl database as follows: **BDNF**: F: AGCGTGTGCGACAGCATTAG, R: GTCCACTGCCGTCTTTTTATCC; **AIF**: F: AGGACTCCTTCCATCACAATGTG, R: TTGGCAAACCCCCTTTCC; **Caspase-3**: F: AGCGCTGAAACAGTATGTTCACA, R: TTCTACTGCTACCTTTCGGTTAACC.

The reference genes (peptidylprolyl isomerase A; (PPIA) and glyceraldehyde 3-phosphate dehydrogenase (GAPDH)) were chosen to standardize all quantitative experiments: **PPIA2**: F: ATA CGG GTC CTGGCA TCT TG, R: AAC TGG GAA CCG TTT GTG TTG; **GAPDH**: F: GGAAGCTTGTCATCAATGGAAAGG, R: ACCAGCATCACCCCATTTGA.

To check the amplification specificity, the qPCR was followed by a melting curve analysis. For each duplicate sample, threshold cycles (Ct) were calculated for BDNF, AIF, caspase-3, PPIA2, and GAPDH genes, and the normalized gene expressions were calculated by the ΔΔCt method. Statistical comparison of qPCR data was performed by comparing the ΔΔCt values of untreated and treated brain samples.

### 4.7. Statistical Analysis

Statistics were calculated using the software package SigmaPlot (v12.0, Systat Software, Chicago, IL, USA) or MATLAB (Mathworks Inc., Natick, MA, USA).

Differences in core temperature, MABP, and HR at each time point were analyzed with two-way ANOVA with repeated measures. Differences in arterial pH, arterial pCO_2_, arterial pO_2_, blood glucose, blood lactate, and delta base excess were compared between the groups at the different time points by two-way ANOVA with repeated measures as well. Comparisons were conducted in each case using Student–Newman–Keuls post hoc test. Data are presented as standard error of the mean (SEM).

All electrophysiological data were analyzed in IBM SPSS 22. For the SpEnt, we used one-way ANOVA with repeated measures; for the PSD and the VEP, we performed two-way ANOVA with repeated measures. Pairwise comparisons were conducted in each case using Tukey’s post hoc test. Data are presented as mean ± SD.

Histological data were analyzed using one-way ANOVA or Kruskal–Wallis analysis of ranks. Pairwise comparisons were conducted in each case using Student–Newman–Keuls post hoc test when appropriate. Data are presented as standard error of the mean (SEM). The observed power of the pairwise comparisons in the different regions was the following: cortex: 0.63, hippocampal CA1: 0.11, CA3: 0.11, caudate nucleus: 0.84, putamen: 0.28, thalamus: 0.12.

Expression of BDNF, AIF, and caspase-3 genes normalized to glyceraldehyde 3-phosphate dehydrogenase (GAPDH) and peptidylprolyl Isomerase A2 (PPIA2) was compared by one-way ANOVA or Kruskal–Wallis analysis of ranks. Pairwise comparisons were conducted in each case using Dunn’s post hoc test.

Statistical significance was defined as *p* < 0.05.

## 5. Conclusions

In newborn pigs, we elicited HIE with experimental asphyxia, leading to neuronal injury that could not be prevented by TH alone. Combination of TH with putative neuroprotective gas therapies such as inhaled molecular H_2_ or CO_2_ to achieve graded restoration of normocapnia did not enhance or complement hypothermia-induced neuroprotection. Based on the findings of the present study, using these gas therapies with these treatment parameters is not recommendable in neuroprotective HIE management.

## Figures and Tables

**Table 1:** Cortical neuronal injury was determined using a neuropathology scoring system based on the occurrence of the most severe pattern observed in 40 visual fields. Higher scores represent more severe neuronal damage. Representative photomicrographs are shown in Supplementary Figure S6.

Score	Morphology of Cortical Damage	Ratio of the Most Severe Pattern per Area
0	No damage	
1	Scattered	<20%
2		21–50%
3		>50%
4	Grouped	<20%
5		21–50%
6		>50%
7	Panlaminar	<20%
8		21–50%
9		>50%

## Supplementary Materials

The following are available online at https://www.mdpi.com/1422-0067/21/18/6801/s1, Table S1: Amplitude-based EEG scoring system, Table S2: Average power spectral density values, Figures S1–S6: Representative photomicrographs from the hippocampus CA1, hippocampus CA3, caudate nucleus, putamen, thalamus and neocortex, respectively.

## Abbreviations

AIF	apoptosis induction factor
Akt	protein kinase B or PKB
A-HT	asphyxia-hypothermia
A-NT	asphyxia-normothermia
BDNF	brain-derived neurotrophic factor
C-NT	control-normothermia
COX-2	cyclooxygenase-2
CREB	cAMP response element binding protein
Ct	threshold cycles
DEPC	diethyl pyrocarbonate
DNA	deoxyribonucleic acid
ECG	electrocardiogram
EEG	electroencephalogram
ERK	extracellular signal-regulated kinase
FFT	fast Fourier transform
GAPDH	glyceraldehyde 3-phosphate dehydrogenase
HI	hypoxia/ischemia
HIE	hypoxic-ischemic encephalopathy
HR	heart rate
InstSpEnt	instantaneous spectral entropy
MABP	mean arterial blood pressure
MAPK	mitogen-activated protein kinase
mRNA	messenger ribonucleic acid
PI-3-K	phosphatidylinositol-3-kinase
PPIA	peptidylprolyl isomerase A or cyclophilin A
PSD	power spectral density
qPCR	quantitative polymerase chain reaction
RNA	ribonucleic acid
RR	respiration rate
SpEnt	spectral entropy
TH	therapeutic hypothermia
TrkB	tyrosine kinase B
VEP	visual evoked potential
