# Supplementary material for: Inhaled H2 or CO2 Do Not Augment the Neuroprotective Effect of Therapeutic Hypothermia in a Severe Neonatal Hypoxic-Ischemic Encephalopathy Piglet Model
Source: Int J Mol Sci. 2020 Sep 16;21(18):6801. doi: 10.3390/ijms21186801 (PMC7555370; doi:10.3390/ijms21186801)
Supplement: Supplementary file 1 [file ijms-21-06801-s001.pdf]

Inhaled H_2 or CO_2 do not augment the neuroprotective effect of therapeutic hypothermia in a severe neonatal hypoxic-ischemic encephalopathy piglet model

Viktória Kovács , Gábor Remzső, Valéria Tóth-Szűki, Viktória Varga, János Németh and Ferenc Domoki

Pattern	***Amplitude based EEG pattern***	***Score***
Continuous	*> 25 μV dominating pattern*	*1*
Discontinuous	*> 25 μV with short durations (1 – 5 s) below*	*2*
Low voltage 1	*25 – 10 μV*	*3*
Low voltage 2	*10 – 15 μV*	*4*
Burst suppression	*Low voltage (< 5μV) with bursts (> 25 μV, 1 – 5 s)*	*5*
Inactive 1	*< 5μV*	*6*
Inactive 2	*practically isoelectric*	*7*
Seizures	*electro/clinical convulsion*	*+2*

Table S1. Amplitude-based scoring system was used for visually evaluation of brain electric activity during asphyxia and the evolution of HIE. High amplitude patterns (> 10 μV) during the initial 10 minute of given time points received lower (1–3) scores, while severely depressed (< 10 μV) brain electric activity was given higher (4–7) ones. The presence of seizure activity during the evaluated period was indicated by adding 2 extra points to the assessed background activity.

		F3	F4	Cz	C3	C4	T3	T4	O1	O2
Delta	C-NT	100.00 ± 14.07	100.00 ± 15.37	100.00 ± 13.39	100.00 ± 12.93	100.00 ± 15.21	100.00 ± 13.30	100.00 ± 16.06	100.00 ± 15.27	100.00 ± 17.01
	A-NT	45.83 ± 4.56	63.51 ± 9.29	47.80 ± 6.05	63.67 ± 10.22	37.23 ± 6.48	43.66 ± 11.27	41.75 ± 7.08	55.41 ± 7.53	38.46 ± 5.79
	A-HT	22.16 ± 5.44	32.02 ± 7.54	41.41 ± 8.71	19.52 ± 3.98	16.83 ± 3.48	9.04 ± 1.93	14.08 ± 5.69	19.32 ± 5.19	24.28 ± 6.22
	A-HT+H_2	36.30 ± 19.42	49.06 ± 19.66	39.84 ± 11.24	38.01 ± 18.71	25.34 ± 27.70	24.86 ± 6.06	35.99 ± 28.41	35.09 ± 9.75	27.16 ± 15.05
	A-HT+CO_2	31.99 ± 6.97	38.22 ± 6.64	41.31 ± 7.57	27.48 ± 7.62	23.95 ± 5.20	13.42 ± 3.14	16.37 ± 3.31	17.15 ± 4.04	21.37 ± 5.28
Theta	C-NT	100.00 ± 18.40	100.00 ± 15.91	100.00 ± 21.33	100.00 ± 19.32	100.00 ± 17.90	100.00 ± 16.12	100.00 ± 24.09	100.00 ± 18.15	100.00 ± 29.48
	A-NT	44.65 ± 6.18	46.12 ± 7.48	45.28 ± 9.52	46.17 ± 6.80	31.64 ± 3.81	39.93 ± 9.65	32.74 ± 6.35	51.98 ± 8.29	32.61 ± 4.07
	A-HT	21.19 ± 5.96	23.60 ± 4.82	33.61 ± 9.08	18.56 ± 3.22	19.79 ± 4.32	11.92 ± 2.45	14.14 ± 3.16	18.48 ± 4.05	21.20 ± 4.28
	A-HT+H_2	36.83 ± 8.18	50.16 ± 32.43	36.72 ± 8.11	28.64 ± 4.21	21.61 ± 6.24	23.45 ± 4.52	24.38 ± 5.60	27.90 ± 4.39	22.46 ± 20.61
	A-HT+CO_2	28.65 ± 7.33	26.54 ± 6.18	33.25 ± 8.59	15.81 ± 4.56	16.48 ± 4.14	10.17 ± 2.69	12.24 ± 3.23	13.81 ± 3.43	15.21 ± 3.96
Alpha	C-NT	100.00 ± 20.23	100.00 ± 20.64	100.00 ± 18.45	100.00 ± 19.87	100.00 ± 16.38	100.00 ± 17.46	100.00 ± 21.29	100.00 ± 15.65	100.00 ± 25.96
	A-NT	35.66 ± 4.61	40.70 ± 6.25	38.52 ± 6.96	45.43 ± 9.23	29.09 ± 3.74	35.02 ± 6.33	32.91 ± 5.43	56.56 ± 11.85	32.74 ± 4.60
	A-HT	21.68 ± 5.48	24.48 ± 5.53	26.07 ± 5.72	17.43 ± 3.16	19.03 ± 3.42	15.12 ± 2.60	17.73 ± 3.82	15.54 ± 3.26	21.02 ± 4.08
	A-HT+H_2	34.63 ± 7.07	40.18 ± 12.03	28.37 ± 4.94	27.57 ± 3.98	21.13 ± 3.45	25.47 ± 4.81	26.80 ± 5.47	33.18 ± 5.76	19.53 ± 3.29
	A-HT+CO_2	22.03 ± 7.33	22.58 ± 9.36	18.50 ± 3.68	11.48 ± 2.79	11.87 ± 3.03	10.80 ± 2.71	11.26 ± 2.95	11.53 ± 2.67	12.47 ± 2.74
Beta	C-NT	100.00 ± 16.79	100.00 ± 15.97	100.00 ± 15.17	100.00 ± 16.51	100.00 ± 15.55	100.00 ± 12.07	100.00 ± 16.15	100.00 ± 12.26	100.00 ± 19.39
	A-NT	41.34 ± 4.89	44.95 ± 5.44	32.47 ± 4.96	49.97 ± 7.57	33.20 ± 4.06	36.67 ± 5.14	39.40 ± 4.68	58.22 ± 8.31	37.01 ± 4.24
	A-HT	37.82 ± 13.00	44.42 ± 14.94	34.08 ± 9.07	25.06 ± 6.52	26.13 ± 6.76	25.66 ± 7.52	32.94 ± 9.76	26.20 ± 7.62	34.45 ± 7.12
	A-HT+H_2	36.03 ± 6.37	38.47 ± 7.40	24.97 ± 4.92	33.22 ± 5.82	24.55 ± 3.98	31.86 ± 4.89	34.84 ± 5.29	45.31 ± 7.61	28.09 ± 4.75
	A-HT+CO_2	32.90 ± 14.75	34.41 ± 15.14	24.29 ± 10.64	19.39 ± 7.81	19.05 ± 8.19	19.93 ± 8.85	24.27 ± 11.83	26.73 ± 7.19	26.45 ± 8.39

Table S2. Average power spectral density (PSD) values in the 5 different groups, containing the frequency and channel information as well. Mean differences were significant (mean±SD, $p<0.05$) the values that were not different form each other are indicated with blue colors. Control-normothermia C-NT, asphyxia-normothermia A-NT, asphyxia-hypothermia A-HT, A-HT+H_2, and A-HT+CO_2.

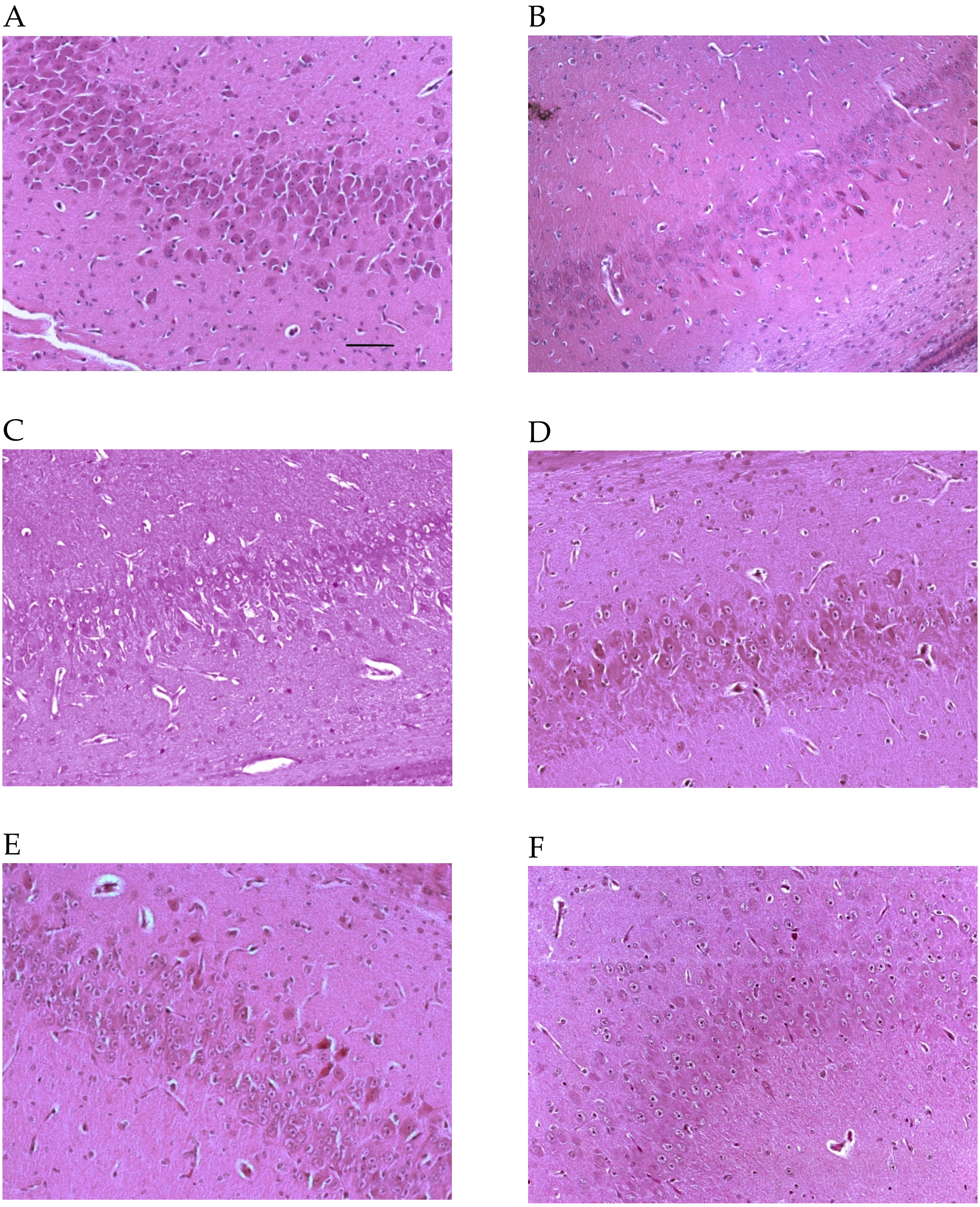

Figure S1. Observed neuronal injury at 48h after asphyxia. Representative H&E stained photomicrographs (20x) show the damaged neurons in the hippocampal CA1 region in the (A), Naïve (B) Control-normothermia C-NT, (C) asphyxia-normothermia A-NT, (D) asphyxia-hypothermia A-HT, (E) A-HT+H_2 and (F) A-HT+CO_2 groups (scale bar: 100μm).

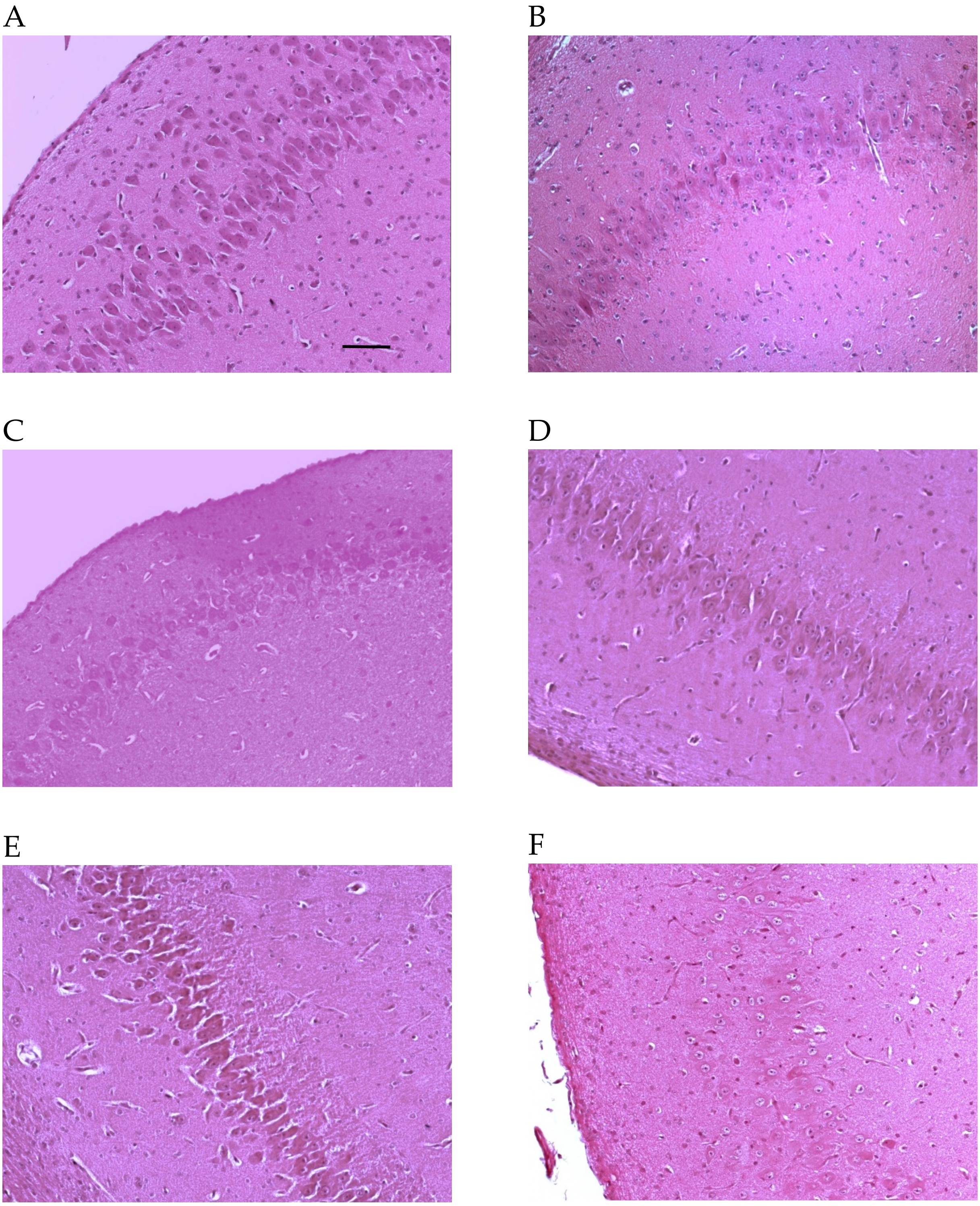

Figure S2. Observed neuronal injury at 48h after asphyxia. Representative H&E stained photomicrographs (20x) show the damaged neurons in the hippocampal CA3 region in the (A), Naïve (B) Control-normothermia C-NT, (C) asphyxia-normothermia A-NT, (D) asphyxia-hypothermia A-HT, (E) A-HT+H_2 and (F) A-HT+CO_2 groups (scale bar: 100μm).

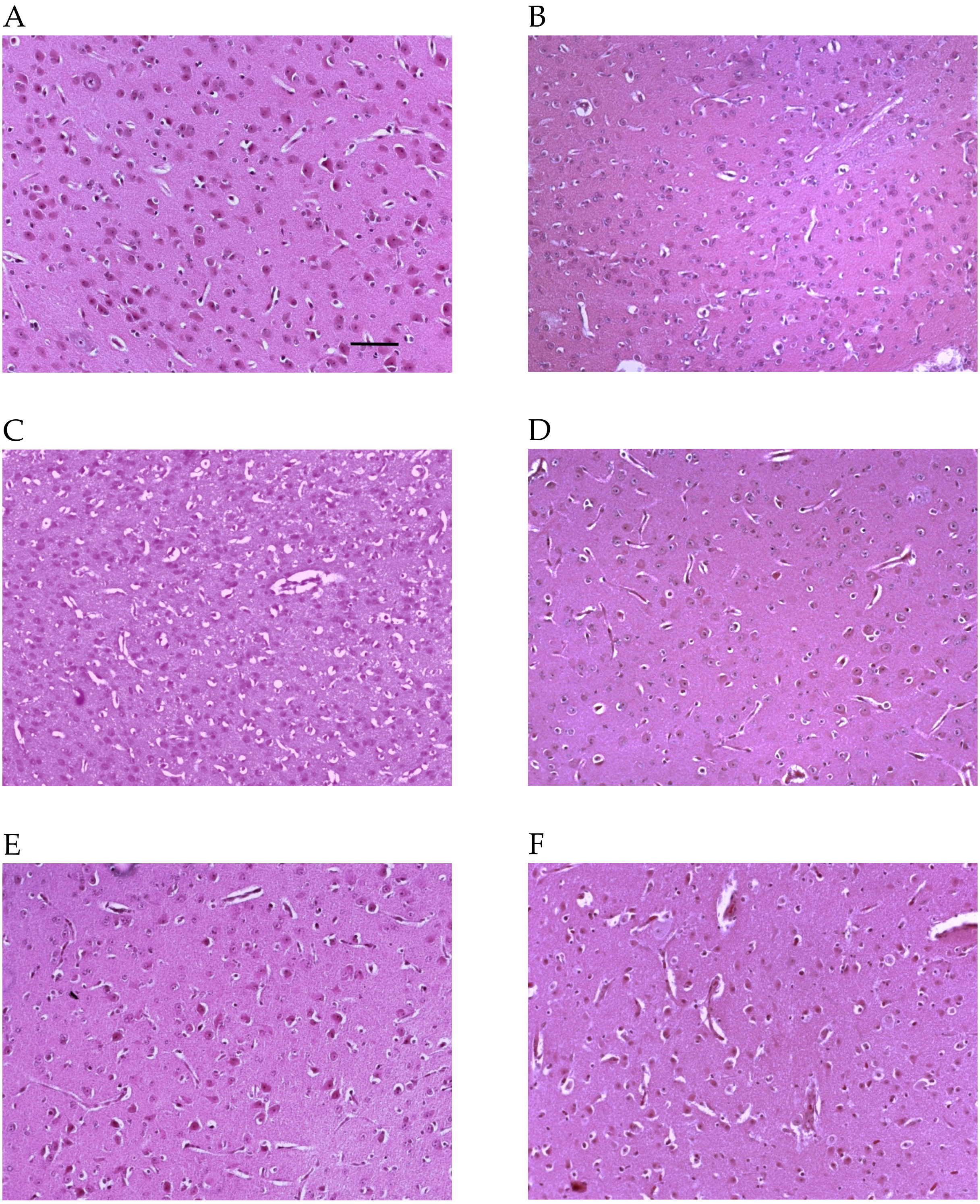

Figure S3. Observed neuronal injury at 48 hours after asphyxia. Representative H&E stained photomicrographs (20x) show the damaged neurons in the caudate nucleus in the (A), Naïve (B) Control-normothermia C-NT, (C) asphyxia-normothermia A-NT, (D) asphyxia-hypothermia A-HT, (E) A-HT+H_2 and (F) A-HT+CO_2 groups (scale bar: 100µm).

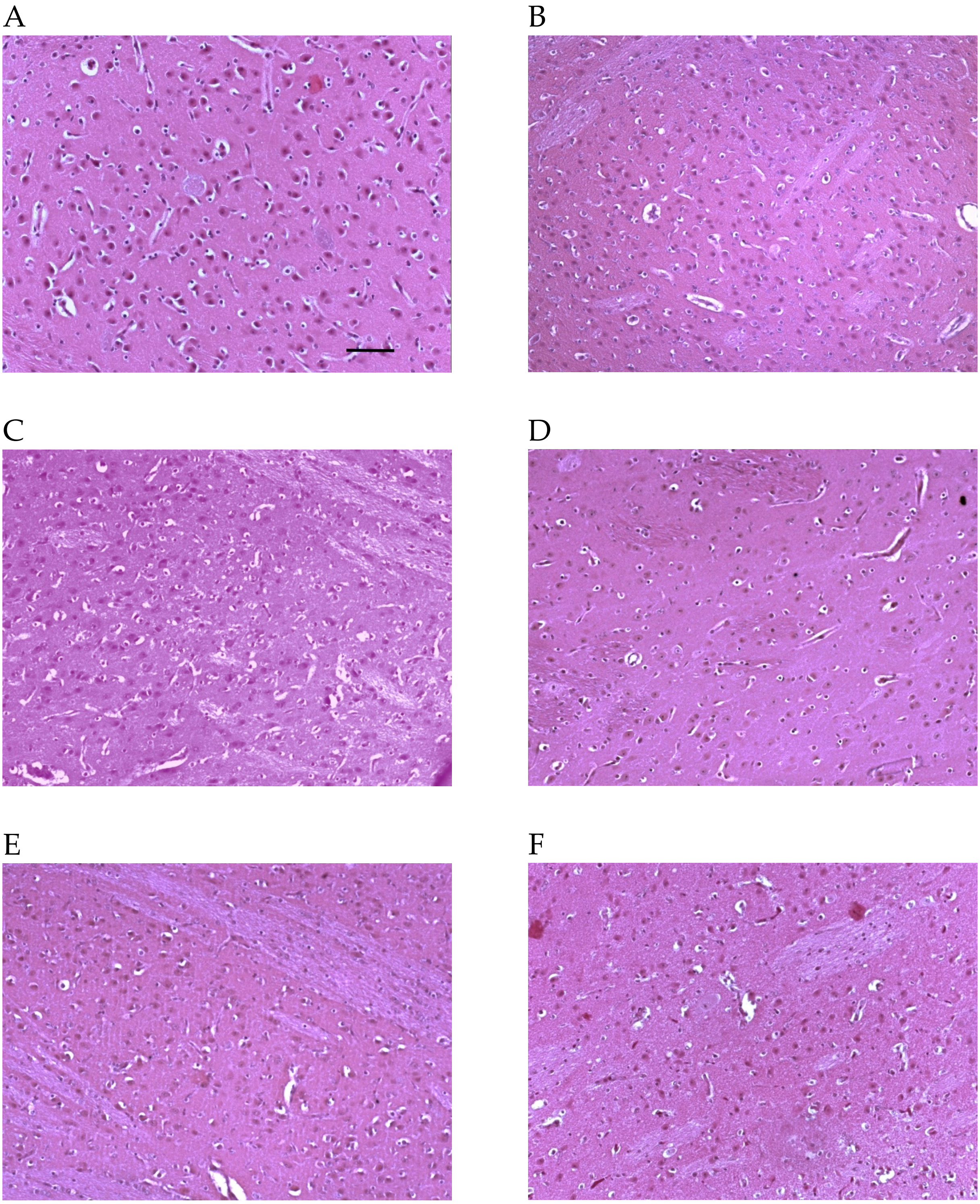

Figure S4. Observed neuronal injury at 48 hours after asphyxia. Representative H&E stained photomicrographs (20x) show the damaged neurons in the putamen in the (A), Naïve (B) Control-normothermia C-NT, (C) asphyxia-normothermia A-NT, (D) asphyxia-hypothermia A-HT, (E) A-HT+H_2 and (F) A-HT+CO_2 groups (scale bar: 100μm).

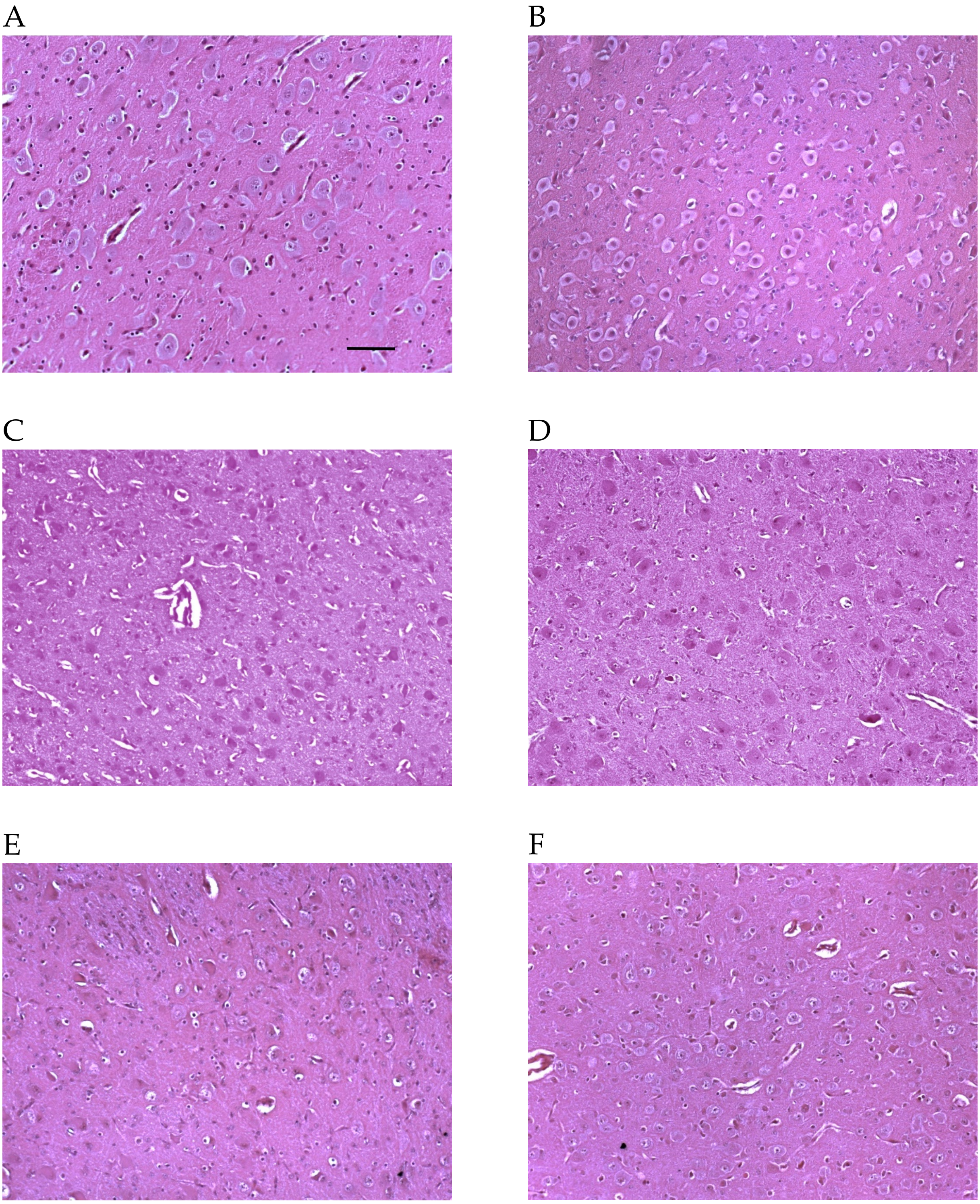

Figure S5. Observed neuronal injury at 48h after asphyxia. Representative H&E stained photomicrographs (20x) show the damaged neurons in the thalamus in the (A), Naïve (B) Control-normothermia C-NT, (C) asphyxia-normothermia A-NT, (D) asphyxia-hypothermia A-HT, (E) A-HT+H_2 and (F) A-HT+CO_2 groups (scale bar: 100μm).

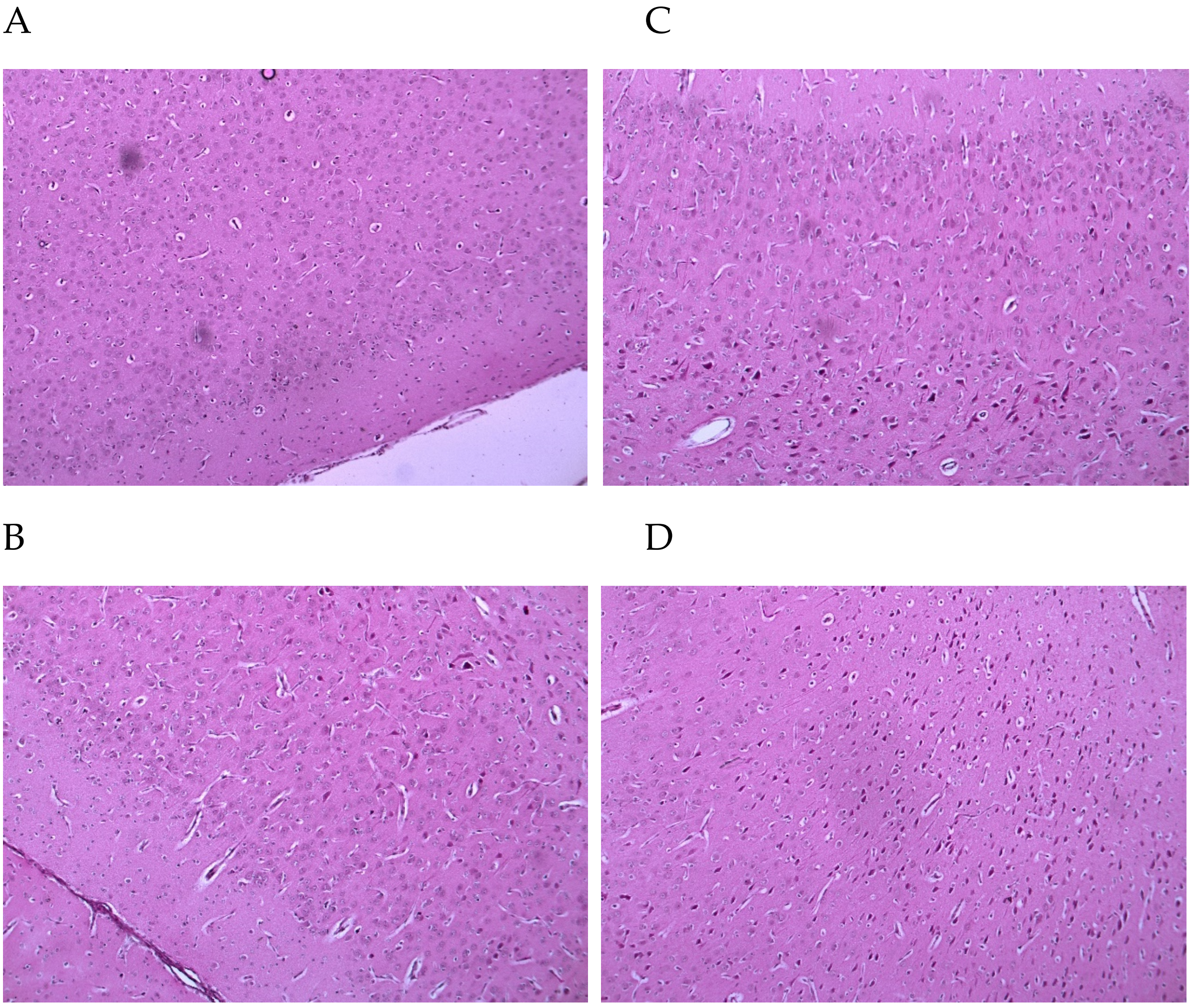

Figure S6. Representative H&E photomicrographs (10x). Cortical neuronal injuries were determined by using a neuropathology scoring system. The observed patterns were the following: (A) Intact cortex, (B) scattered -, (C) laminar -, (D) confluent neuronal damages.
